# 1286. Evaluating Dalbavancin Suppression Therapy in Patients at High Risk for Recurrent Infections

**DOI:** 10.1093/ofid/ofad500.1125

**Published:** 2023-11-27

**Authors:** Michael Shaw, Caroline Derrick, Morgan Pizzuti, Julie Ann Justo, Jeremy J Frens, Wesley D Kufel, Daniela de Lima Corvino, Tamara Krekel, Salam Kabbani, Jeff Brock, Jon Derringer, Ricardo M La Hoz, Patrick M Kinn, Beatriz Diaz, P Brandon Bookstaver, Ana Cristina Antolí

**Affiliations:** Prisma Health - Richland, Columbia, South Carolina; University of South Carolina School of Medicine, Columbia, South Carolina; Prisma Health Richland - University of South Carolina, Columbia, South Carolina; Dartmouth Hitchcock Medical Center; Cone Health, GREENSBORO, North Carolina; Binghamton University School of Pharmacy Sciences, Binghamton, New York; University of Alabama in Birmingham, Vestavia, Alabama; Barnes-Jewish Hospital, St. Louis, MO; Olathe Medical Center, Olathe, Kansas; MercyOne Medical Center, Des Moines, Iowa; Baptist Health Medical Center, Little Rock, Arkansas; University of Texas Southwestern Medical Center, Dallas, TX; University of Iowa Hospitals & Clinics, Iowa City, Iowa; Hospital Universitario La Paz, Madrid, Madrid, Spain; Prisma Health Richland - University of South Carolina, Columbia, South Carolina; Hospital Nuestra Señora de Sonsoles, Ávila, Castilla y Leon, Spain

## Abstract

**Background:**

Dalbavancin is a long-acting lipoglycopeptide used off-label in the treatment of deep-seated infections. An emerging potential niche for dalbavancin is chronic suppressive therapy in patients with retained sources. Limited data exists describing the use of DST. The purpose of this study is to evaluate the long-term safety, effectiveness, and optimal dosage regimens of dalbavancin suppression therapy (DST).

**Methods:**

This was a multicenter, multinational, retrospective observational cohort study among adult patients who received as least 1 dose of suppressive dalbavancin between January 1, 2018 and March 31, 2023. The primary endpoint was the proportion of patients on DST who experienced treatment-emergent adverse events (TEAEs). Additional endpoints included assessment of clinical failure defined as hospitalization or microbiological recurrence due to index organism a and a detailed assessment of dosing regimens used.

**Results:**

A total of 46 patients who received DST were assessed. Therapy was continued for an average of 6.8 months. The majority of patients (83%) had a retained prosthesis or device. *Staphylococcal spp.* was the most prevalent pathogen (47%) and the majority were monomicrobial. TEAEs were observed in 18 (39%) patients. Nephrotoxicity was the most common TEAE with 15 (33%) patients experiencing an AKI during DST. Only 1 TEAE (2.2%) was considered related to dalbavancin therapy TEAEs that led to the discontinuation or dosing modification of DST occurred in 1 (2.2%) patient. The most frequently used dosage scheme was 1500 mg IV every 2 weeks, followed by 1000 mg IV every 4 weeks. Eight patients (17%) were hospitalized or had microbiological recurrence due to the index organism, occurring an average of 7 months after starting DST.

**Conclusion:**

DST was utilized for a variety of infections, most of which involved prostheses. Though TEAEs were observed in nearly 40% of patients, those resulting in discontinuation or dose modification were rare despite long-term follow-up. Majority of patients experienced clinical success at an average 7-month follow-up. Future prospective studies should assess pharmacokinetics of DST to determine the optimal dosage regimen.

**Disclosures:**

**Julie Ann Justo, PharmD, MS, FIDSA, BCPS**, Gilead Sciences: Advisor/Consultant|Shionogi: Advisor/Consultant|Vaxart: Stocks/Bonds **Wesley D. Kufel, Pharm.D., BCPS, BCIDP, AAHIVP**, Merck & Co.: Grant/Research Support **Tamara Krekel, PharmD, BCPS, BCIDP**, AbbVie: Honoraria|Merck: Honoraria|Shionogi: Honoraria **Ricardo M. La Hoz, MD**, Takeda: Advisor/Consultant

